# Do Young Women Need Treatment for Hypertension After Pregnancy Complications?

**DOI:** 10.1161/JAHA.118.009159

**Published:** 2018-05-13

**Authors:** Christina Y. L. Aye, Einas Elmahi, Henry Boardman, Paul Leeson

**Affiliations:** ^1^ Oxford Cardiovascular Clinical Research Facility Department of Cardiovascular Medicine John Radcliffe Hospital University of Oxford United Kingdom; ^2^ Nuffield Department of Women's and Reproductive Health John Radcliffe Hospital University of Oxford United Kingdom

**Keywords:** Editorials, preeclampsia/pregnancy, prevention, Cardiovascular Disease, Women, Primary Prevention, Pregnancy

## Introduction

Pregnancy complications, such as hypertensive disorders of pregnancy, are well established as independent risk factors for cardiovascular diseases in later life in both the mother^1^, ^2^ and the offspring.^3^, ^4^ Studies of women from 1 to 40 years after childbirth consistently show increased rates of hypertension, ischemic heart disease, cerebrovascular disease, and cardiovascular mortality following a hypertensive pregnancy.^1^, ^2^ There is also evidence of a dose response according to the severity of hypertension during pregnancy. The risk of cardiac disease following gestational hypertension is associated with lower risk compared with early onset preeclampsia, particularly when complicated by preterm delivery.^2^

In this issue of the *Journal of the American Heart Association* (*JAHA*), Egeland et al add a real‐world perspective of this risk by reporting findings on rate of medication use for hypertension over the 10 years after pregnancy. Importantly, these women were normotensive before pregnancy; therefore, the findings reflect new‐onset disease.^5^ The authors also consider other possible reasons for developing hypertension. They show that a pregnancy complication is the main explanation for medication use in more than a quarter of the young women being treated. These findings are based on a substantial Norwegian cohort of >60 000 women that was linked to the Norwegian Prescription Database. In agreement with previous studies, a hypertensive pregnancy in addition to a very preterm delivery, before 32 weeks of gestation, was associated with the highest hazard ratio for subsequent hypertension (age‐adjusted hazard ratio: 14.33; 95% confidence interval, 9.03–22.70) compared with pregnancies without hypertension.

An explanation for the association between pregnancy complications, such as hypertensive disorders of pregnancy, and long‐term cardiovascular disease has been that they are expressions of the same disease process. This is supported by the fact that they share common risk factors such as high maternal age, diabetes mellitus (both prepregnancy and gestational), obesity, and renal disease.^6^, ^7^, ^8^, ^9^, ^10^, ^11^, ^12^ Mothers who develop complications may have an adverse underlying cardiovascular phenotype that could predate the affected pregnancy and that may deteriorate further during the acute systemic disturbance of the pregnancy complication.^13^ Defining this relationship is complex: Retrospective analysis is not without limitations, and adequately powered longitudinal studies from before conception to after childbirth are limited.^14^ Nevertheless, there appear to be links among prepregnancy blood pressure levels, hypertension during pregnancy, and later problems. A rare study of >3000 women found that ≈50% of differences in blood pressure several years after pregnancy were explained by blood pressure differences before pregnancy.^15^

Simply attributing later cardiovascular risk to standard cardiovascular risk profiles in women who have pregnancy complications is, however, probably overly simplistic. These women appear to have distinct risk characteristics,^16^ including low age, autoimmune disease, nulliparity, or an increased interval between pregnancies (>10 years), that alter risk for pregnancy complications. In addition, multiple pregnancy, ethnicity (nonwhite), assisted conception, and change in paternity are relevant, suggesting that distinct genetic and immunological components may drive early risk. Egeland et al found a persistent excess risk of hypertension after adjusting for numerous prepregnancy and postpartum risk factors, and in analyses restricted to women with a healthy prepregnancy body mass index, hazard ratios observed were similar to those in the whole study population.^5^ An impact on fetal growth was also not required because small size for gestational age was, on its own, not a risk factor for hypertension. Consequently, pregnancy appears to be a “stress test” for a specific type of cardiovascular risk associated with earlier onset disease in seemingly healthy individuals at time of pregnancy.

In summary, this study by Egeland et al provides further evidence that common pregnancy complications, such as a hypertensive pregnancy and preterm birth, identify women at higher risk of cardiovascular disease in later life. A major strength of the work is that it provides real‐world clinical relevance, reinforcing the message that hypertension risk after pregnancy is not benign but sufficient to warrant treatment within relatively short time periods. Furthermore, more than a quarter of the risk of needing medication is attributable to pregnancy complication alone rather than to any other identifiable risk factors. Pregnancy complications occur relatively early women's lives. Consequently, the presence of a pregnancy complication provides an opportunity to identify high‐risk women early and to offer primary prevention advice and intervention before end‐stage disease has become established.^17^ Studies aimed at understanding the mechanisms behind this increased risk may reveal novel targets that will have future benefit.

## Disclosures

None.
